# Case Report: Unusual presentation and atypical course of a case of ureterocolic fistula after anterior resection for sigmoid cancer

**DOI:** 10.3389/fonc.2025.1549485

**Published:** 2025-06-11

**Authors:** Salvatore Tramontano, Biancamaria Iacone, Valentina Parrella, Antonio Gargiulo, Anna Tedesco, Umberto Bracale

**Affiliations:** ^1^ Department of Physics, University of Salerno, Salerno, Italy; ^2^ Department of General and Emergency Surgery, Ospedali Riuniti San Giovanni di Dio e Ruggi d’Aragona, Salerno, Italy; ^3^ Department of General Surgery, University of Naples Federico II, Naples, Italy

**Keywords:** ureterocolic fistula, colorectal cancer, surgical complications, ureteral injury, ureter

## Abstract

The ureterocolic fistula (UF) can be a rare but serious complication of abdomino-pelvic surgery, gynecological procedures, oncological or inflammatory conditions and, especially in colorectal surgery, it can be due to anastomotic leaks or iatrogenic injuries of the ureter. Treatment is multidisciplinary, often involving endoscopic urological procedures or surgery, when necessary. We present a case of an UF following laparoscopic anterior resection for sigmoid cancer Some peculiar topics, like an early clinical presentation and a rapid resolution with adequate approach, are very interesting and offer good example for suspicion of UF and management. Our patient presented fever and watery diarrhea in 12^th^ POD. CT scan was positive for contrast leakage between the third-low of the left ureter and the rectum. Fistula solved with endoscopic and percutaneous approach, with no need of surgical treatment, thanks to multidisciplinary approach and early treatment.

## Introduction

Ureterocolic fistula (UF) represents a rare but highly concerning complication of colorectal surgery (1, 2). The pathogenesis and presentation patterns are highly variable, as reported in the literature (2–4). It may be associated with colorectal anastomosis or recent inflammatory events (diverticulitis, inflammatory bowel diseases etc.) (4–7): in the first case, an anastomotic leak generally leads to adhesive local process and infiltration of the ureter, most often at the middle-lower third of the ureter; in the second case, the inflammatory process can involve the ureter along its entire length, resulting in a leak of the anastomosis (8). Clinical presentation varies, and symptoms are often latent. Generally, pneumaturia and fecaluria are observed, but the most distinctive symptom is the presence of urine in the stool, which leads to watery diarrhea, often accompanied by pain (7, 9). Treatment is multidisciplinary and almost always requires an endoscopic urological approach, combined with surgical treatment, when necessary (2, 7, 10, 11). Medical/endoscopic treatment is effective in about half of cases, but often can be required the surgical reconstruction of both the urinary tract and colon.

We present a case of UF following laparoscopic anterior resection for sigmoid cancer, managed in our Operative Unit. The distinctive features of our case report are early clinical presentation from the surgery and rapid clinical resolution of symptoms after treatment.

SCARE criteria were followed for adequate explanation of the report (12). For comprehensive evaluation, PROCESS checklist was completed (13).

## Case presentation

We present the case of a 61-year-old man, BMI 23, with an endoscopic diagnosis of adenocarcinoma of the sigmoid colon, which was incidentally discovered during diagnostic investigations for respiratory issues. Patient had no significant previous clinical history, no smoking anamnesis and no familiarity for any type of cancer; furthermore, he was not taking any medications for any medical condition.

Preoperative staging CT did not identify any metastases or signs of locoregional infiltration of the disease. Therefore, the patient underwent laparoscopic anterior resection with colorectal anastomosis according to the Knight-Griffen technique.

The postoperative course was regular, with normal bowel function, early resumption of oral feeding and discharge on the fifth postoperative day (POD). Histological examination confirmed adenocarcinoma of the sigmoid colon, with positive lymph node removal (pT4a G3 N2a according to TNM/AJCC 8^th^ ed.). The postoperative follow-up had been regular.

## Diagnostic assessment

**Figure 1:**
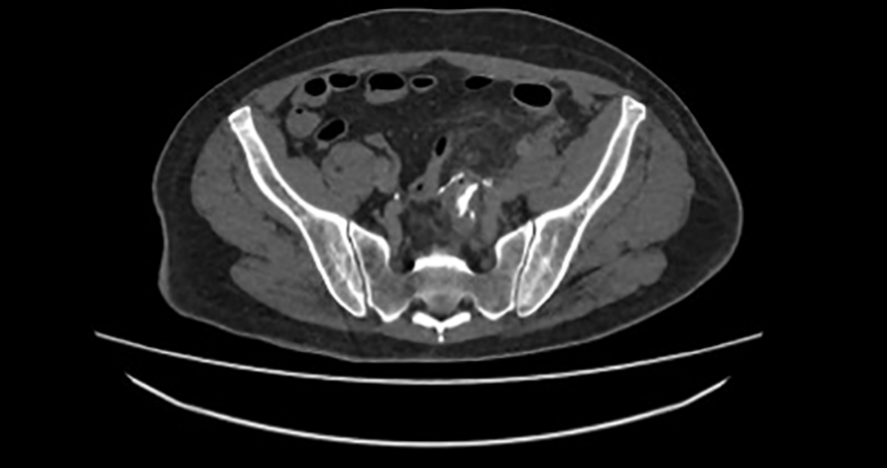
Passage of iodate contrast from urinary tract to rectum.

On the 12^th^ POD the patient reported abdominal pain, fever and diarrhea, with regular urine output. The diarrhea was described as watery and associated with abdominal pain. Due to this condition, he went to the Emergency room of our Hospital. The blood tests showed WBC 13000, hemoglobin 11.2 g/dL, creatinine 0.8 mg/dL, eGFR 0.98 ml/min, CRP 4.3 mg/dL and 39°C body temperature. CT scan showed, in tardive and ultratardive phases, the passage of urine into the rectum (**Figure 1**). Throughout the patient’s observation, there were no signs of fecaluria or passage of air during urination, although the CT scan showed air in the urinary bladder.

**Figure 2:**
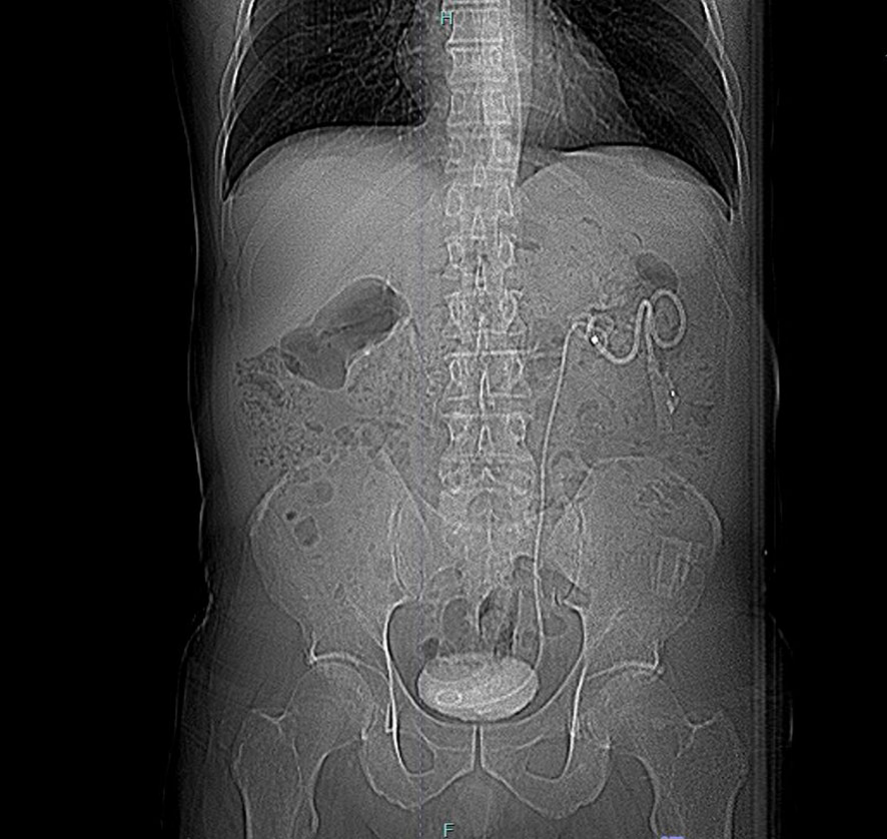
Descending pyelography and creation of nephrostomy with stent positioning.

Suspecting an UF, retrograde pyelography was performed, but the ureter could not be identified above the affected segment, likely due to kinking near the fistula. Consequently, on the 15th POD, an anterograde pyelography was conducted, identifying the leak and an ureteral stent was placed, with nephrostomy (**Figure 2**). This procedure was difficult because hydronefrosis was lacking, but percutaneous approach was successful.

After the procedure, the patient’s bowel movements and urine output (from both nephrostomy and catheter) normalized, resolving the sepsis. Urine culture and hemoculture were negative, and the patient remained afebrile for the entire subsequent hospital stay. The urinary catheter was removed on the third day from stenting, with regularization of urine output.

At subsequent follow-up, one month after stenting, the CT scan did not document any contrast medium leakage from the urinary tract, neither into the intestinal tract. The ureter appeared normal, with good urine drainage from the stent into the bladder. The removal of the stent and of nephrostomy was scheduled 40 days after placement. The patient was asymptomatic and began the adjuvant treatment recommended by the oncologist 60 days after colic resection. At the last follow-up, six months after the intervention, the patient was in good clinical condition and asymptomatic.

## Patient perspective

UF is a complex and often underestimated complication in colorectal surgery. Early treatment can avoid hard urological reconstructive surgery and delays in the continuation of oncological therapies. Our experience of early resolution with multidisciplinary treatment confirms the need for a multidisciplinary approach that diverts urinary drainage away from the leak site.

## Discussion

This case report represents a rare occurrence of a serious complication of colorectal surgery. UF, especially when involving an anastomotic segment, is associated with worse prognosis, delays in the initiation of adjuvant therapy, and, in over one-third of cases, the need for surgical revision with reimplantation of the ureter (14, 15). A multidisciplinary, interventional, and endoscopic approach can be effective, especially if the diagnosis is early, as it reduces the risk of fibrosis in the ureteral segment, which can prevent adequate functional recovery (16). In some cases, the leak is so extensive that it leads to the passage of enteric material in the urine, posing a high risk of sepsis. For these reasons, the need for nephrostomy placement for several months is very common, particularly in oncological patients at high risk of sepsis (3).

Moreover, causes of ureteral fistula include urinary tract calculi, iatrogenic trauma, diverticulitis, radiation, cancer, and tuberculosis (17). The first cause reported by literature is nephrolithiasis, often when occurred obstruction and pyelonephritis. A fistulous tract can develop in any area affected by chronic inflammation, necrosis, or ischemia (18). Colorectal surgery is an infrequent cause of UF, that is important recognize to obtain the best solution. An analysis of ureteral injuries in colorectal surgery over a 10-year period in the United States reported an incidence of 0.28%, on over two million of case analyzed (4). A French multicentric retrospective cohort study, through experience of GRECCAR group reported an incidence of 0.32% (19). In fact, majority of reports described generic ureteral injury after colorectal surgery, while only few authors specifically indicated UF after surgery, a little part of all ureteral injuries. This is frequently associated to patients with inflammatory bowel disease and to obstetric patients, also evidenced by metanalysis of Yanagisawa (20). On this way, the largest American report on complications after colorectal surgery also confirmed rate of ureteral injury (1.0%), but did not report specific rate of colorectal fistula, that remains very infrequent in all reports (21). Recently, two reports described cases of iatrogenic fistula in pediatric patients (22, 23).

From a pathophysiological perspective, while diverticular UF have a clear inflammatory pathogenesis, fistulas from anastomoses likely involve multiple factors: there may be damage to the ureter wall, leading to an inflammatory reaction and probably a microleak of urine that is not detected at the time of surgery (24). Adhesions to the anastomotic site can lead to adhesive phenomena, potentially resulting in the creation of a direct fistulous tract. Conversely, a primary anastomotic leak can cause local damage to the ureter, creating the leak without local abscess formation (10).

This report analyzes technical approach in diagnostic and therapeutic steps. This is useful because data of literature on detection of UF are lacking, like indicated by literature review. All surgeons, especially colorectal specialists, should learn to approach this occurrence.

Despite the improvements achieved with intraoperative techniques, such as indocyanine green visualization (25) and magnified vision of robotic approach (26), incidence of ureteral injury is not reduced. Among these, a smaller proportion leads to the formation of UF. For this reason, integrated management of this complication must always be considered. We have not considered technical points to prevent ureteral injury, because our case focused diagnostic and therapeutic approach to UF. Moreover, some steps, identified correctly by Yellinek (27), should be stressed. Understanding of the anatomical course of the ureters and the adjacent organs is necessary in every lower abdominal surgery. Identification and reidentification of the ureter during each step of the dissection is a common lesson for colorectal surgeons. Also, in cases of inflammation or severe fibrosis, the ureter should be isolated in an unaffected area. The ureter may be displaced from its usual course in these cases. Many authors agree that preoperative stent placement did not ensure intraoperative identification of injury (27), as indicated by data of Palaniappa in his comparative study on a large cohort (1).

The peculiarities of our report, compared to data in the literature on UF, involve three main aspects: early detection, absence of typical symptoms (fecaluria), and relatively rapid resolution. Regarding diagnosis, the time frame for the pathogenesis of a complex fistula is generally longer, especially due to the ureteral caliper, that requires more time for the formation of the fistula (8, 11). Stenosis, due to direct injury or compromised vascularization of the ureter, is much more common, typically occurring within the first ten days after surgery (19). Our experience confirms that clinical suspicion should be heightened in the presence of fever and irregular bowel movements. The presence of urine in the stool should always be considered in cases of watery diarrhea, especially if it is colorless or yellowish (14).

Finally, regarding the resolution of the leak, literature data indicate that reconstructive surgery is required in over 30% of cases, with months of stent maintenance and nephrostomy drainage (6, 15). This approach leads to healing within 6-12 months in a variable percentage of cases, reported in literature until 60% (28, 29). Our choice of a combined approach, avoiding surgical revision, has proven to be rapidly effective in controlling symptoms and infection. The decision to remove the stent is also reported in literature as a means to assess the patency of the fistulous tract, which is sometimes covered by the stent and can lead to false negatives (15). Literature also reports instances of stent exchanges, prior to healing or surgical intervention (24).

The decision for intestinal diversion, noted by various authors, seems to be crucial only when the passage of feces into the urinary tract is significant, particularly in extensive leaks involving the ureter (19). These are the cases where the repair process is also compromised, and conservative treatments serve only as a bridge to surgical reconstruction of the ureter into the bladder (4, 19). Early identification of these cases can improve surgical timing. The choice of a loop colostomy or terminal colostomy with removal of the anastomotic complex is reported in the literature, especially in the presence of pericolic abscesses or extensive tissue loss at the anastomotic and ureteral site (29). Clinical symptoms and worsening of the condition often indicate the need for surgical exploration and possible intestinal diversion.

## Data Availability

The raw data supporting the conclusions of this article will be made available by the authors, without undue reservation.
